# Measuring Energy Expenditure in Sub-Adult and Hatchling Sea Turtles via Accelerometry

**DOI:** 10.1371/journal.pone.0022311

**Published:** 2011-08-04

**Authors:** Lewis G. Halsey, T. Todd Jones, David R. Jones, Nikolai Liebsch, David T. Booth

**Affiliations:** 1 Department of Life Sciences, Roehampton University, London, United Kingdom; 2 Joint Institute for Marine and Atmospheric Research, University of Hawaii at Manoa, NOAA - Kewalo Research Facility, Honolulu, Hawaii, United States of America; 3 Zoology Animal Care, University of British Columbia, Vancouver, British Columbia, Canada; 4 Queensland Brain Institute, University of Queensland, St Lucia, Queensland, Australia; 5 Physiological Ecology Group, School of Biological Sciences, University of Queensland, St Lucia, Queensland, Australia; Institut Pluridisciplinaire Hubert Curien, France

## Abstract

Measuring the metabolic of sea turtles is fundamental to understanding their ecology yet the presently available methods are limited. Accelerometry is a relatively new technique for estimating metabolic rate that has shown promise with a number of species but its utility with air-breathing divers is not yet established. The present study undertakes laboratory experiments to investigate whether rate of oxygen uptake (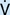 o_2_) at the surface in active sub-adult green turtles *Chelonia mydas* and hatchling loggerhead turtles *Caretta caretta* correlates with overall dynamic body acceleration (ODBA), a derivative of acceleration used as a proxy for metabolic rate. Six green turtles (25–44 kg) and two loggerhead turtles (20 g) were instrumented with tri-axial acceleration logging devices and placed singly into a respirometry chamber. The green turtles were able to submerge freely within a 1.5 m deep tank and the loggerhead turtles were tethered in water 16 cm deep so that they swam at the surface. A significant prediction equation for mean 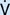 o_2_ over an hour in a green turtle from measures of ODBA and mean flipper length (R^2^ = 0.56) returned a mean estimate error across turtles of 8.0%. The range of temperatures used in the green turtle experiments (22–30°C) had only a small effect on 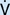 o_2_. A 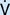 o_2_-ODBA equation for the loggerhead hatchling data was also significant (R^2^ = 0.67). Together these data indicate the potential of the accelerometry technique for estimating energy expenditure in sea turtles, which may have important applications in sea turtle diving ecology, and also in conservation such as assessing turtle survival times when trapped underwater in fishing nets.

## Introduction

Measuring metabolic rate (energy expenditure) of sea turtles is difficult. There are logistical problems in applying time-energy budgets [1] to these species, because for example the construction of time–energy budgets requires detailed observations of behaviour in the wild and replication of observed activities in the laboratory while simultaneously measuring energy expenditure through indirect calorimetry [2]. Values obtained using the doubly-labelled water (DLW) technique [3] can sometimes be spurious due mainly to the high water flux and low rate of carbon dioxide production of turtles [2], [4]. While in some species the DLW technique is valid during feeding periods [5] (but not during fasting [2]), it only provides a single value for mean metabolic rate over the experimental period. Yet data on the metabolic rates of free-ranging sea turtles, particularly at a range of temporal resolutions, are important for understanding the physiology of these animals, their population dynamics and, in turn, managing their conservation (Wallace and Jones 2008).

There are a number of methods for estimating energy expenditure in animals that should be applicable to sea turtles. The carapace makes turtles particularly suitable for instrumenting with data loggers (e.g. [6]), affording the opportunity to measure a pertinent physiological or behavioural proxy of energy expenditure. While heart rate has been successfully measured in free-ranging sea turtles using data loggers [7] and in captive individuals using a transmitter or transducer [8], [9], it has not yet been tested as a method for estimating their metabolic rate. The accelerometry technique for estimating energy expenditure involves measuring the body motion of animals after calibrating the magnitude of body motion with rate of energy expenditure. The metric of body motion typically employed is termed ‘overall dynamic body acceleration’ (ODBA) [10] and is derived from recordings of acceleration in the three spatial dimensions by a data logger placed on a fixed point of the animal. Significant relationships between energy expenditure and ODBA in terrestrial laboratory settings have been published for ten endotherm species [10], [11], [12], [13], [14] and three ectotherm species [15], [16], [17].

In contrast, Halsey et al. [18] argue that the three studies to date that have measured energy expenditure and derivatives of acceleration in air breathing, semi aquatic species [18], [19], [20] have not provided evidence that these variables correlate during activity in water. Given that sea turtles spend the majority of their lives at sea, it may not be possible to calibrate ODBA with metabolic rate. As discussed in Halsey et al. [18], one possible reason for this lack of a correlation in air-breathing aquatic animals is that they do not consume the amount of oxygen between dives equal to the amount of oxygen being consumed in the dive [21]. An uncoupling between oxygen uptake and oxygen consumption might be particularly acute in sea turtles for several reasons. Their diving behaviour is highly variable sometimes including long periods of rest or even sleep underwater [22], [23]. Further, they exhibit pulmonary shunting [24], adjust buoyancy through changes in respiratory gas volume [25], [26] and possibly undertake non-pulmonary respiration.

However, other reasons put forward for the lack of a relationship between metabolic rate and ODBA include that movements of the animal are damped during periods of submergence resulting in activity during periods at the surface having an unrepresentatively large effect on ODBA [18], and that heat loss during submergence renders energy expenditure independent of body motion [27], [28], [29]. Juvenile and adult sea turtles surface rarely [30] and with only their head emerging to the air, thus essentially they remain in one medium while at sea. Furthermore, they are committed to the oceans, rarely returning to land, so their metabolic rate is not affected by a change in rate of heat loss when they enter the water to start diving. In addition, as ectotherms the temperature of their tissues approximates to a few degrees above ambient temperature [31], though active tissues can be considerably higher [32], and thus when ambient temperature is constant their metabolic rate is less thermally dependent than can be the case for endotherms.

Thus while there is a need for a new and suitable method for estimating the metabolic rate of turtles at sea, it is unclear whether the accelerometry technique represents a valid solution. Using laboratory experiments, the present study investigates whether it is possible to correlate rate of oxygen uptake at the surface in submerging and/or swimming turtles with ODBA; if relationships exist this would suggest applicability of the accelerometry technique for estimating energy expenditure in turtles at sea.

## Materials and Methods

The ethical guidelines of the Association for the Study of Animal Behaviour were followed during the present study. This study was approved by the University of British Columbia Animal Care Committee (Animal Care Protocol A10-0012) and The University of Queensland Animal Ethics Committee (AEC approval number: SBS/481/09). In 2003, green turtles *Chelonia mydas* used in the present study were imported from the Cayman Turtle Farm (Grand Cayman, British West Indies, CITES Export Permit 2002/ky/000112) to the Zoology Animal Care Centre, Department of Zoology, University of British Columbia (CITES Import Permit CA02CWIM0129). Hatchling loggerhead turtles used in the present study were collected and released in January 2010 under Queensland Government Environmental Protection Agency Departmental Authorisation issued to Col Limpus who manages the scientific research programs at the Mon Repos Turtle Rookery.

### Green turtles

The turtles were held in a large oval fibre glass tank (10×3×1.5 m deep) filled with seawater except when they were kept in isolation (isolation tanks) for an experiment, during which they were instrumented with an acceleration logger. The water in the large tank was heated and water temperature was set between 22 and 30°C inclusive during the course of the experiments. These temperatures were chosen because the majority of green turtle populations experience year-round sub surface temperatures in the mid-twenties [5], [33]. Further details concerning the long term care of the turtles are provided in [2]. Six of the available healthy turtles were used in the present study (mean mass: 35.5 kg; mass range: 25.4 to 43.6 kg). Three of them were male, while the gender of the other three is unknown.

### Loggerhead turtles

Two turtles (19.4 and 20.8 g), which had just hatched onto a beach at Mon Repos, Australia, were placed in a respirometer chamber (34×28×19 cm deep) for several hours during which time they swam almost continuously, while instrumented with an accelerometer sensor. Air temperature ranged between 26 and 28°C and water temperature was maintained at 28°C.

### Respirometry – green turtles

The animals were fasted for two weeks before the start of the experiments, to ensure that they were completely post absorptive [34]. The isolation tank (1.5×1×1.5 m deep), filled with seawater, was covered by an acrylic respirometry dome to trap expired gases. The temperature of the seawater always matched the seawater temperature in the large oval tank, and turtles were exposed to each temperature, within the large tank, for a minimum of five hours before experiments. Each turtle was used in either two or three experiments, each experiment being at a different temperature. The turtles were able to move around in the isolation tank in three dimensions and quickly learnt to breathe into the respirometry dome. While it is possible that the turtles suffered some degree of stress when first placed into the chamber, the first few minutes of data were not recorded. Furthermore, stress has a negligible effect on metabolic rate and in contrast to the heart rate technique the measurements recorded by the accelerometry technique are not significantly influenced by stress levels [35]. A gas mix of 20.94% O_2_, remainder N_2_, was forced through the respirometry dome at 14.5 L min^−1^ and the exhaust sampled at 400 ml min^−1^ using an O_2_ analyser (Applied Electrochemistry S-3A, AEI Technologies) after having been dried and purged of CO_2_ to avoid dilution of O_2_ in the sample gas [36]. The volume of the air space in the respirometry dome was ∼16 L, the time constant of the system (time to reach a 95% step change) was therefore ∼2.7 min, and the lag time of the respirometer and tubing was ∼10 s. Further details of the respirometry setup are provided in [2]. Data from the O_2_ analyser were recorded at a frequency of 0.5 Hz and later analysed using commercial software (DataCan V Data Acquisition and Analysis Software, Sable Systems International). Measurements were taken over the course of the experiment, during which the turtles typically exhibited a variety of behaviours including consistent swimming below the water surface, manoeuvring in all three dimensions, and resting both near the surface and at the bottom of the tank. Despite the relatively confined space, the turtles rarely knocked against the sides of the tank after the first couple of minutes of each experiment. Each experiment lasted about one hour and directly afterwards the turtles were returned to the large oval tank.

After baseline correcting for analyser drift, the data trace of fractional concentration of O_2_ against time was converted into a trace of rate of O_2_ uptake (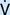 o_2_) against time using the following equation:
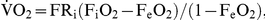
where FR_i_ = flow rate into the respirometry dome; F_i_O_2_ = incurrent fractional concentration of O_2_; F_e_O_2_ = excurrent fractional concentration of O_2_. The trace was then inverted and the area underneath the trace to the baseline integrated to calculate volume of oxygen uptake.

### Respirometry – loggerhead turtles

The respirometry setup used was a negative pressure flow-through design, identical to that reported in detail by Booth [37]. In brief, the turtles had an accelometer sensor (details below) glued to the dorsal surface of their carapace, and the five 0.05 mm diameter copper wires connecting the accelometer to recording equipment were woven into a single cord that tethered the hatchling in the respirometry tank (34×28×19 cm high). They swam in seawater at 28°C, 16 cm deep, for 2–3 hours. The tank was painted black except on one side, where a low intensity light was placed to induce directional swimming. The tank was sealed with a lid using vacuum grease, creating an air space of 1.5 L. Two 2-mm holes were drilled in the lid, one at the front corner to allow a continuous incurrent of air, the second at the back corner diagonally opposite, which was connected to a tube that sampled air at 90–100 mL/min. The tether's length was adjusted so that the hatchling could swim freely but rarely touched the walls or bottom of the tank. Air drawn from the air space above the water, passed through a drierite® water absorber, a mass-flow meter (FMA 0–100 mL/min Omega.com, Omega Engineering Inc., Stamford, CT, USA), an oxygen analyser (PA-1B Sable Systems, Las Vegas, NV, USA) and then a pump before being vented to the atmosphere. The oxygen analyser was calibrated before and after measurements with dried room air. Signals from the oxygen analyser and flow meter were sampled every 30 s. To apply an instantaneous correction to metabolic rate [38] the wash out characteristics of the respirometry system were measured by flushing the system with nitrogen and monitoring the rate at which the oxygen concentration changed when room air was drawn into the system. These data were used to calculate the system's effective volume. Oxygen uptake was calculated using equation 11.2 of Lighton [39] assuming a respiratory quotient of 0.8 after the washout correction was applied.

### Accelerometry – green turtles

The turtles were instrumented with a triaxial acceleration data logger. The loggers used were set to record tri-axial acceleration (0–2 *g*) at 10 Hz, using a custom-made interface program for PCs. The loggers had 22-bit resolution and recorded data onto a 2 Gb RA memory card. This recording frequency is sufficiently high when using measures of acceleration primarily as a proxy for energy expenditure [14]. The loggers and 3.6 V lithium battery used as their power source were housed in a two-stage cylindrical waterproof casing (80 mm length, 23 mm greatest diameter).

A bed for the logger (90×53×12 mm deep), was custom made of acrylic glass. The upper side included a curved groove within which the logger sat while the underside created a flat surface for gluing to the carapace of the turtle using superglue (cyanoacrylate cement). The logger was strapped to the upper side of the bed using two cable ties. Grooves across the width of the underside of the bed stopped lateral movement in the ties. The bed was attached to the shell, with the length along the antero-posterior axis, on a relatively flat section of scutes towards the posterior end and as close to the midline as possible (the carapace of some green turtles is flat across the midpoint of the posterior portion, whereas in other individuals it is ridged in which case the logger was positioned slightly laterally). The total weight of the attachment onto the turtle was 44 g, which represented between 0.1 and 0.2% of body mass.

Data from the acceleration loggers were downloaded onto a PC via a card reader. The x axis of the logger measured sway, the y axis measured surge, and the z axis measured heave (see [40] for more details). From the downloaded logger data an approximation of absolute *g* resulting from only dynamic acceleration in each of the three dimensions was extracted from each axis following removal of the static acceleration using a running mean [14], with a width of 2 s as recommended in [41]. These values were then summed to produce ODBA (see [10] for more details).

### Accelerometry – loggerhead turtles

The hatchlings were equipped with a triaxial acceleration sensor (Model MMA7260Q, Freescale Semiconductor, Arizona, USA, set to the 2 g range and to record at 40 Hz) wired to a power supply and Power Lab data acquisition system (ADInstruments, New Zealand). The sensor was attached dorsally to the mid-line at the level of the front flippers with superglue (cyanoacrylate cement). The sensor was 6×6×3 mm deep and weighed 2 g, about 10% of the body mass of the hatchlings. Similarly to the green turtles, dynamic acceleration in each of the three dimensions was extracted, again using a running mean with a width of 2 s, and these values then summed.

### Data analysis – green turtles

The relationship between mean 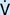 o_2_ and ODBA, separately for each turtle and experiment, was investigated for the same data averaged at either each consecutive 2.5 min, 5 min, or 10 min period. This comparison of between temporal resolutions assessed a trade-off between the number of data points input to a regression along with the range in values they represented, and the amount of scatter in those data points. While averaging 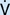 o_2_ and ODBA across shorter periods provided more data points and encompassed a slightly greater range of values, only at 10 min periods were the strength of the relationships consistently high. Therefore, further analysis was undertaken only on mean 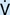 o_2_ and ODBA calculated for each consecutive 10 min period of the experiments, which provided five or six data points per experiment. Several morphometric variables (body mass, carapace length, mean flipper length) were recorded for each turtle. Preliminary analyses of the data were undertaken using Excel (Microsoft Corp.) with further analyses conducted using JMP (v. 7, SAS Institute Inc.) and Minitab (v. 16, Minitab Inc.).

For each individual experiment the data were analysed using a single linear regression to check that the relationship was significant. Then, general linear models (GLMs), using standard least squares regression, were generated to investigate the relationship between 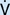 o_2_, ODBA, temperature and several morphometric variables (all represented as covariates). These models also included a categorical variable that identified each turtle (‘Turtle ID’) to recognise that the data are repeated measures within each turtle. Firstly, a mixed linear effects model (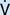 o_2_ ∼ ODBA + Temperature + Turtle ID{random} + ODBA * Temperature + ODBA * Turtle ID + Temperature * Turtle ID) tested for a relationship between 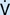 o_2_ and ODBA, and whether this relationship varied between turtles and with temperature. The model was then run without the Turtle ID interaction terms to generate a 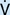 o_2_ prediction equation for green turtles where morphometric variables are unknown, which included ODBA, Turtle ID and, if a significant predictor, temperature. To provide information on the accuracy of this prediction equation, the 95% confidence intervals and the relative standard errors of the estimate (the relative size of the error associated with predicted 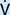 o_2_) were calculated for values of ODBA within the range recorded in the present experiments. When applied to a new set of green turtles, because these measures are affected by not only the predictive power of the prediction equation but also the number of turtles and ODBA data points input to the equation, they were calculated for three feasible scenarios in terms of the number of turtles used and amount of data collected. For more information and details of the calculations employed, see Green et al. [42].

The accuracy of this prediction equation was further assessed through a validation, which provides quantified estimate errors that do not necessarily relate strongly to R^2^. The same data used to derive a prediction equation can be reasonably employed to validate that equation using a jack-knife statistical technique [13], [15], [43]. The principle is that for each individual turtle, values of 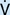 o_2_ are estimated from ODBA recorded during an experiment for that turtle, using a common prediction equation generated from the data for all turtles apart from the individual turtle of interest. These 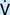 o_2_ estimates are then compared with the values of 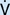 o_2_ measured concurrently with ODBA for that turtle. In the present study, for each individual turtle separately, mean 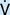 o_2_ and mean ODBA were calculated for one experiment (representing one temperature, which was randomly selected while ensuring a full range of temperatures was represented across the turtles). Then, mean 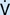 o_2_ was predicted from mean ODBA, based on the common prediction equation. Actual and predicted mean 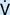 o_2_ for each turtle were then used to calculate the mean algebraic error (calculated as [observed 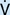 o_2_ – estimated 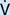 o_2_]/observed 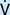 o_2_×100) across all individuals. Due to the duration of the experiments, this validation assessed predictive accuracy for when 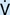 o_2_ was estimated over about an hour.

Some individual variation in the 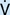 o_2_-ODBA relationship is likely to be due to differences in body mass and/or morphometrics. However, inclusion of morphometrics in a prediction equation is not straightforward if those values are fixed for each individual animal, which is typically the case. One option is to regress the individual estimate for each animal associated with the first prediction model against morphometric measures and include the relationship in the 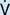 o_2_ prediction equation [44], however this can reasonably be done only for a single morphometric variable. In the present study, the individual estimates were significantly related to body mass (R^2^ = 0.40) and mean flipper length (R^2^ = 0.42) but not to carapace length. Body mass was significantly and strongly related to flipper length (R^2^ = 0.91) and the latter is generally easier to measure than the former, particularly in the field. Therefore, a second 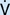 o_2_ prediction equation was generated by adding to the first prediction equation a linear description of individual variance by mean flipper length. A validation of this equation was then conducted, as described earlier.

### Data analysis – loggerhead turtles

Mean 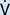 o_2_ and ODBA were calculated for each consecutive 1 min period during experiments. For each turtle individually, the data were analysed using a single linear regression to check that the relationship was significant. The data for both turtles were then input to a mixed effects linear model to predict 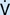 o_2_, which included turtle ID as a random factor. The analysis of the loggerhead turtle data was undertaken only to test whether a relationship between 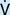 o_2_ and ODBA was present; because data were only obtained for two individuals, while it is suitable to generate an equation to describe a relationship for those two individuals, it is not reasonable for such a relationship to be offered or tested as a prediction equation for other individual hatchlings.

## Results

The 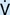 o_2_ data for the present study compliment those reported by Southwood et al. [45] for green turtles of slightly smaller mean mass in the field (32.5 kg) and by Jones et al. [2] for green turtles in the laboratory with a mean mass of 22 kg. In Southwood et al. [45] these individuals averaged about 16 ml O_2_ min^−1^ over several hours at 26°C where activity levels were low while in Jones et al. [2] they averaged 11 ml O_2_ min^−1^ at 25°C in a 20-day fasted state, again over several hours, where resting was the dominant activity. In the present study 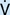 o_2_ averaged 54 ml O_2_ min^−1^ and the turtles were typically fairly active during the trials.

Histograms of 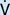 o_2_ and ODBA for all experiments together indicated that these variables were reasonably normally distributed. All single linear regressions of 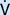 o_2_ against ODBA for each individual green turtle at each temperature to which they were exposed were significant and returned a mean R^2^ of 0.74 (range: 0.29 to 0.98).

All data were included in all GLM analyses. A mixed effects linear model (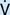 o_2_ ∼ ODBA + Temperature + Turtle ID{random} + ODBA*Temperature + ODBA*ID + Temperature*ID; R^2^ = 0.68) indicated a significant relationship between 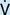 o_2_ and ODBA (P<0.001). There was also an interaction between ODBA and turtle ID (P<0.003) indicating that the slopes of the 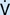 o_2_-ODBA relationships varied among individual animals. There was no effect of temperature (P = 0.241) or an interaction between ODBA and temperature (P = 0.684) or temperature and turtle ID (P = 0.112). Thus temperature was not an important factor in describing 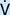 o_2_ in this analysis. The same model with the main effect of temperature and the interaction terms removed represents the prediction equation for use with green turtles when morphometric information (mean flipper length) is not available:
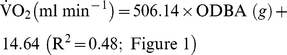
(1)


Validation of this equation returned a mean algebraic error of 8.3% (range: −8.4 to 28.9%). The 95% confidence intervals and relative standard errors of the mean are graphically represented in Fig. 2.

The equation generated for predicting 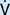 o_2_ of green turtles from ODBA and mean flipper length is:
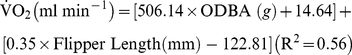
(2)


Validation of this equation returned a mean algebraic error of 8.0% (range: −21.5 to 26.4%).

Histograms of 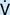 o_2_ and ODBA for both of the loggerhead turtle hatchlings individually indicated that these variables were reasonably normally distributed. Although the data for one of the two individuals is arguably non-linear, this is the single case out of eight sea turtles analysed in the present study and therefore for consistency and simplicity the loggerhead turtle data were analysed using a mixed effects linear model (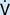 o_2_ ∼ ODBA + Turtle ID{random}). ODBA was a significant variable and the model returned an R^2^ of 0.67 (Fig. 3).

## Discussion

**Figure 1:**
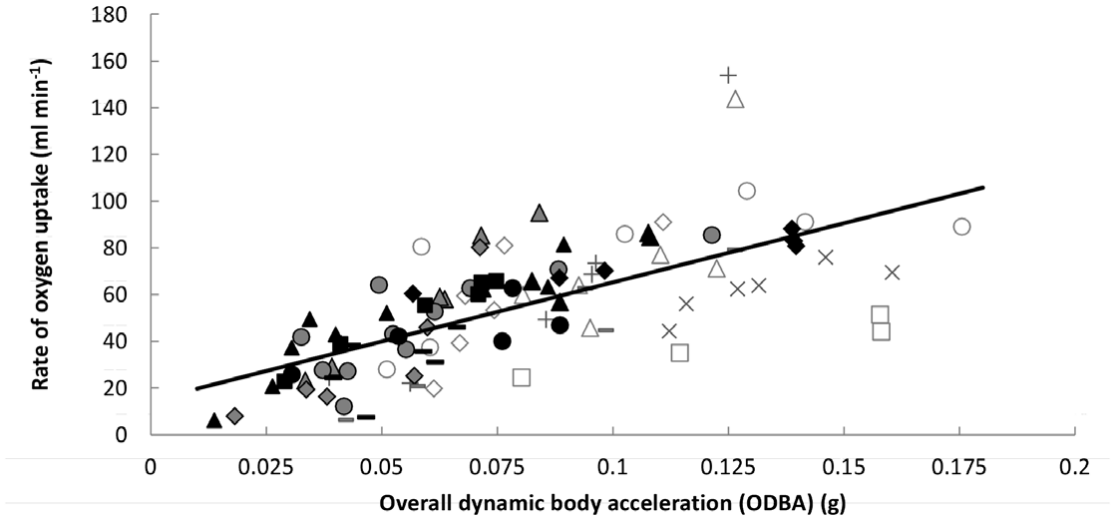
Rate of oxygen uptake against ODBA at 10 min intervals for six green turtles during a range of activity levels and temperatures. Each type of symbol represents data points for a particular individual. Darker versions of symbols denote higher ambient water temperatures (closed unfilled symbols represent temperatures between and including 22 and 25°C, unclosed symbols 25°C, closed grey-filled symbols 26 and 27°C, and closed black-filled symbols 29 and 30°C). Values for rate of oxygen uptake are shown adjusted for inter-individual differences between turtles (by subtracting from each data point the parameter estimate for Turtle ID obtained from a linear mixed effects model; see Methods). The common slope shown, derived from the linear mixed effects model, is described by 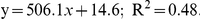

**Figure 2:**
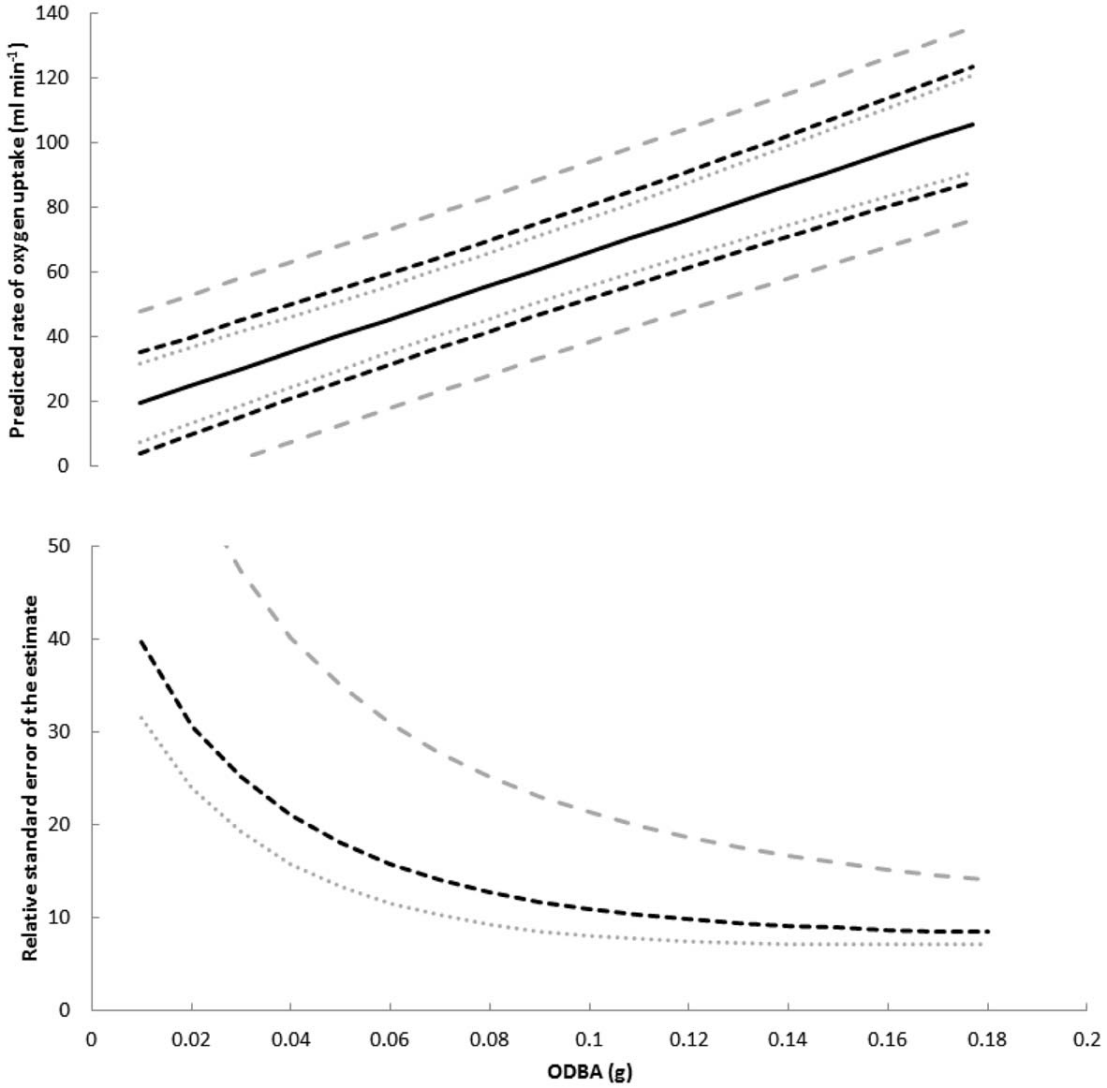
Simulation demonstrating the accuracy of predictions of rate of oxygen uptake from ODBA in green turtles using prediction **equation 1**, at different values of ODBA, for different sampling scenarios. The three scenarios are: (1) six turtles measured for one hour each; black dashed lines, (2) one turtle measured for one hour; grey dashed line, (3) 20 turtles measured for one week each; grey stippled lines. In each case, each 10 min period of data is averaged. (a) The 95% confidence intervals. The black solid line represents prediction equation 1 (see Fig. 1). (b) The relative standard error of the estimate ( = SEE/predicted rate of oxygen uptake×100).

**Figure 3:**
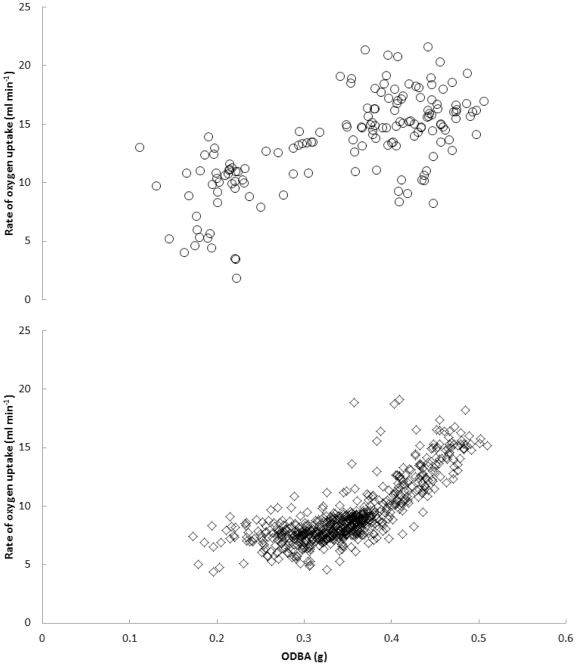
Rate of oxygen uptake against ODBA at one minute intervals for two loggerhead turtle (denoted by the two symbol types) *Caretta caretta* hatchlings while swimming during the first few hours after emerging from the egg. The common slope for these two turtles, derived from a linear mixed effects model, is described by 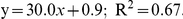

Regressions of 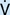 o_2_ against ODBA for the six green turtle sub-adults and two loggerhead turtle hatchlings in water were significant (Figs. 1 and 3) and therefore demonstrate the potential for the accelerometry technique to be a useful method for estimating the metabolic rate of sea turtles in the wild. The mean algebraic errors associated with the green turtle prediction equations based on the regression analyses are comparable to previous studies using ODBA for estimating mean 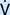 o_2_ for a group of individuals of other species [11], [13]. However, as is typically the case with calibration techniques for estimating metabolic rate [35], [46], the range of algebraic errors associated with the prediction equations generated in the present study are too large to consider those equations as being suitable for estimating energy expenditure of single individuals. Consequently, assessments of accuracy of the prediction equations derived in the present study are made based on combining data for multiple individuals. As is shown in Fig. 2, the accuracy of predictions of 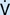 o_2_ is noticeably better when prediction equation 1 is applied to multiple animals and a larger data set. However, at least beyond one individual, this variation is overshadowed by the relationship between the relative size of the estimate errors and mean ODBA; equation 1 is much more accurate at predicting 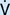 o_2_ for values of ODBA greater than 0.1 (relative standard error of the estimate is around 10%) and indicates that, unsurprisingly, ODBA is a better predictor of 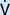 o_2_ when activity levels are greater [11].

If the arguments put forward by Halsey et al. [18] are valid then the present data are the first to provide strong evidence of a relationship between 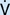 o_2_ and ODBA in an air breathing, diving animal, and this may be explained by some key aspects of sea turtle behaviour and physiology that, in contrast to many diving species, mean that 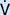 o_2_ and ODBA do not uncouple. First, green turtles do not sit on the water surface between periods of submergence where the 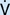 o_2_-ODBA relationship is likely to be very different to that underwater. Second, green turtles rarely experience an increase in rate of heat loss when they start to dive because the majority of their existence is spent at sea rather than including a significant terrestrial component. Also, at ambient temperatures experienced in the wild [5], [33], which approximate to those in the present study, metabolic rate is not thermally dependent [5], [45]. Sea turtles exhibit a sigmoidal relationship between metabolic rate and temperature, which reaches close to a plateau between about 20 and 25°C and likely explains why ambient temperature was not a significant predictor of 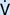 o_2_ in the GLM analyses.

Furthermore, and crucially, by sampling 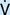 o_2_ over 10-minute periods, measurements of 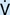 o_2_ likely represent rate of energy expenditure, which does not appear to be the case in this species when 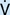 o_2_ is analysed at the resolution of a single surface period. This is because respirometry is only an effective method for indirectly measuring metabolic rate when an animal is in physiological steady state at the whole-animal level [36], i.e. when oxygen uptake across the lung-blood barrier equals oxygen consumption by the cells. However, during diving activity, sea turtles are unlikely to be in steady state for a number of reasons centring around their periodic breathing coupled with the dive response that is evoked during periods of submergence to increase efficiency of oxygen use [7], [47]. Furthermore, surface periods may precede or succeed submergence periods incorporating high activity or rest/sleep, and sea turtles also adjust their buoyancy [25], [26] by adjusting the volume of respiratory gas stores, both at the surface and while submerged. However, by analysing 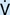 o_2_ data for green turtles over a sufficiently long time frame in the present study, multiple surfacing periods were included over which 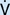 o_2_ averaged out to represent metabolic rate. While there is a relationship between 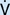 o_2_ per surface period and energy expenditure during that period and the associated dive in some mammalian and avian air-breathing divers [48], [49], these species do not exhibit some of the complicating behaviours of turtles and also tend to breathe at the surface for relatively long periods. Furthermore, 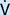 o_2_ per surface period may still underestimate rate of energy expenditure in these species if some of the oxygen consumed during a diving bout is repaid at the end [50], [51].

Sea turtles weigh around 12–50 g at hatching and, depending upon species, reach masses of well over 100 kg after several decades. While the present study has developed prediction equations using ODBA applicable only for turtles between 25 and 45 kg, since these were the masses of the experimental sub-adult turtles available, this study also presents evidence that ODBA can be a valuable derivation for estimating energy expenditure in much smaller individuals (Figure 3). The tethering method used for the respirometry experiments with the loggerhead hatchlings meant that they were able to breathe continuously while swimming which, along with their size and the relatively low variation in their activity, likely explains why it was possible to obtain significant relationships between 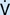 o_2_ and ODBA at the resolution of just one minute. Hatchlings tend to stay close to the surface when swimming in the wild [52] and so tethering them during calibrations provides swimming data representative of their natural state [37], with therefore 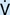 o_2_ and ODBA probably not changing significantly from the non-tethered state. For the sub-adult green turtles, while new calibrations based on data obtained from larger tanks may be preferable for applying ODBA to ascertain energy expenditure during periods of constant swimming such as during migrations, already the present calibrations should be applicable for situations where green turtles are submerged but not swimming consistently or constantly [53], such as when grazing on sea grass [54]. Indeed, there are many examples of 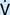 o_2_ proxy calibrations obtained for diving species where the laboratory tank has limited diving behaviour being applied successfully to obtain 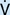 o_2_ estimates for those species in the wild (e.g. [55]).

Measuring the energy expenditure of sea turtles has a number of important applications in terms of understanding their ecology as well as informing their conservation. For instance, a particularly urgent example is ascertaining the survival time of sea turtles trapped in fishing nets (shrimp trawls, pound nets, bottom gillnets). [56] estimated that nearly 900,000 sea turtles interact with U.S. based shrimp fisheries yearly resulting in 80,000 mortalities. While U.S. federal regulation requires turtle excluder devices on all otter trawls, several other trawl types are exempt (e.g., skimmer, butterfly nets, pusher head) being limited by tow time. Similarly, licensed bait shrimpers are regulated by tow time. Presently it is assumed that trapped turtles consume oxygen at resting rates or lower (i.e. by exhibiting the classic dive response) but this is unlikely given that they react to being trapped [57] by greatly increasing flipper beat frequency in attempting to escape. Turtle oxygen storage capacity is known fairly accurately (18–27.4 ml O_2_ kg^−1^ depending upon species; [58], [59]) thus details on rate of energy expenditure while ensnared is extremely important for informing regulations on net submergence durations aimed to reduce trawling-related turtle fatalities.

The present study provides evidence for the validity of the accelerometry technique for estimating the energy expenditure of sea turtles through demonstration of significant relationships between 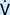 o_2_ and ODBA. While in the past the size of acceleration loggers has limited their potential application to medium and large animals, the technology is advancing rapidly; much smaller loggers are currently under development with the promise of being able to measure and recording ODBA, and thus estimate active metabolic rate, in sea turtles from the time of hatching onwards.
